# Autofluorescence-based tissue characterization enhances clinical prospects of light-sheet-microscopy

**DOI:** 10.1038/s41598-024-67366-2

**Published:** 2024-08-04

**Authors:** Alice M. Jacob, Anna F. Lindemann, Julia Wagenpfeil, Sergej Geiger, Yannik C. Layer, Babak Salam, Sarah Panahabadi, Darius Kurt, Maximilian W. M. Wintergerst, Frank A. Schildberg, Daniel Kuetting, Ulrike I. Attenberger, Zeinab Abdullah, Alexander M. C. Böhner

**Affiliations:** 1 Institute of Molecular Medicine and Experimental Immunology, Medical Faculty, University Hospital Bonn, University of Bonn, Venusberg-Campus 1, 53127 Bonn, Germany; 2 Clinics for Diagnostic and Interventional Radiology, University Hospital Bonn, Venusberg-Campus 1, 53127 Bonn, Germany; 3 Clinic for Diagnostic and Interventional Neuroradiology, University Hospital Bonn, Venusberg-Campus 1, 53127 Bonn, Germany; 4 Department of Ophthalmology, University Hospital Bonn, Venusberg-Campus 1, 53127 Bonn, Germany; 5 Clinic for Orthopedics and Trauma Surgery, University Hospital Bonn, Venusberg-Campus 1, 53127 Bonn, Germany; 6 Department of Biomedical Imaging and Image-Guided Therapy, Medical University of Vienna and General Hospital, Waehringer Guertel 18-20, 1090 Vienna, Austria

**Keywords:** Translational research, Light-sheet microscopy, Fluorescence spectroscopy, 3-D reconstruction

## Abstract

Light sheet fluorescence microscopy (LSFM) is a transformative imaging method that enables the visualization of non-dissected specimen in real-time 3D. Optical clearing of tissues is essential for LSFM, typically employing toxic solvents. Here, we test the applicability of a non-hazardous alternative, ethyl cinnamate (ECi). We comprehensively characterized autofluorescence (AF) spectra in diverse murine tissues—ocular globe, knee, and liver—employing LSFM under various excitation wavelengths (405–785 nm) to test the feasibility of unstained samples for diagnostic purposes, in particular regarding percutaneous biopsies, as they constitute to most harvested type of tissue sample in clinical routine. Ocular globe structures were best discerned with 640 nm excitation. Knee tissue showed complex variation in AF spectra variation influenced by tissue depth and structure. Liver exhibited a unique AF pattern, likely linked to vasculature. Hepatic tissue samples were used to demonstrate the compatibility of our protocol for antibody staining. Furthermore, we employed machine learning to augment raw images and segment liver structures based on AF spectra. Radiologists rated representative samples transferred to the clinical assessment software. Learning-generated images scored highest in quality. Additionally, we investigated an actual murine biopsy. Our study pioneers the application of AF spectra for tissue characterization and diagnostic potential of optically cleared unstained percutaneous biopsies, contributing to the clinical translation of LSFM.

## Introduction

Light sheet fluorescence microscopy (LSFM) is a powerful imaging technique enabling real-time 3D visualization of undissected specimens. To achieve this, the sample is selectively illuminated in a thin plane, giving rise to the name “light sheet microscopy.” However, LSFM requires optical clearing of the samples to render them transparent, facilitating light penetration through the tissue^1^. Current optical clearing methods, which often aim at large samples like entire human organs, mostly involve toxic solvents, such as Benzyl Alcohol/Benzyl Benzoate (BABB) or Dibenzyl Ether, which are hazardous. The sample preparation protocols can also be highly time-consuming, ranging from several days to weeks^2–4^. Instead of relying on highly volatile, toxic and in some cases explosive chemicals, optical clearing can also be performed using the non-hazardous Ethyl Cinnamate (ECi). ECi-based protocols offer several advantages, including low cost and reduced safety requirements, making them more accessible and widely available^5,6^. However, experts have noted that ECi may result in slightly less optimal tissue transparency compared to traditional methods^7^. In general, LSFM is usually applied to answer highly specific questions in several areas of biological science, such as developmental biology or botany, using a variety of fluorescent tracers, fluorescence labeled markers, and/or animal reporter lines^8^.

While LSFM has predominantly been applied to address specific questions in basic biological sciences, there is growing interest in its potential translation into clinical settings, such as prostate biopsies^9^. Human percutaneous biopsies are the most widely used approach to obtain a tissue sample from a living patient. Utilizing specifically designed tools, a tissue cylinder with a diameter of ca. 1 mm and a length of up to 15 mm can be harvested from medically relevant structures, e.g. a liver lesion suspicious to be a malignant tumor. In clinical routine, biopsies are fixated and systematically dissected for investigation via 2D microscopy. This conventional technique usually involves the staining with hematoxylin & eosin or other compounds^10^, depending on the medical question at hand. Alternatively, fluorescence microscopy of thin tissue sections is available as a more elaborate method allowing to address more specific questions^11^. In most modern studies, autofluorescence (AF) is considered a fluctuating, and in essence disruptive, factor. In consequence, several studies detail procedures to dampen or overcome AF^12–14^.

What, however, has not been attempted to our knowledge, is the structural 3D-characterization of tissues based solely on their AF spectra with non-toxic reagents.

Physically, AF occurs as the excitation light is absorbed and emitted in a specific spectrum of light other than the excitation light^15^. In organic chemistry, AF is observed particularly in covalent bounds in π-configuration as in cyclic or planar aromatic molecules^16,17^. Historically, microscopy studies relying on AF were mostly carried out for plants^18^ as most plant cells incorporate compounds with a high fluorescent efficiency, e.g. chlorophyll^19^. Furthermore, some marine species display strong bioluminescence^20^, greatly facilitating their investigation using similar microscopy equipment. In medicine, several biochemical compounds, e.g. aromatic amino acids, collagen or nicotinamide-adenine dinucleotide (NAD), exhibit AF capabilities^21,22^, which can in some cases be also amplified with the formaldehyde used for tissue fixation^23^, the commonly used agent for tissue preservation and fixation in clinical routine^24^.

Medical AF investigations have been conducted only a handful of times, relying on BABB protocols, looking at the development of mouse testes and human skin biopsies^25,26^. Leveraging AF data in conjunction with established staining panels could offer novel diagnostic parameters.

In this study, we sought to encompass a diverse range of tissues, including the liver, ocular globe with neural tissue, lacrimal gland and knee joint, to analyze their AF spectra establishing a general protocol optimized for simplicity in usage and cost-efficiency. Our approach not only involves characterizing tissues based on AF spectra but also employs the obtained data of hepatic tissue as ground truth for machine learning segmentation tools. With our study, we aim to contribute to the potential clinical translation of LSFM for tissue characterization and diagnostic applications.

## Results

### Standardizing optical clearing and image acquisition protocols in light sheet fluorescence microscopy

In order to robustly image and quantify a broad variety of tissues we optimized a clearing protocol suitable for all parenchymal organs and musculoskeletal structures. Our pipeline prioritized practicability, work safety and cost efficiency. Based on ethyl cinnamate, we simplified existing protocols significantly: after animal sacrifice and EDTA perfusion, whole organisms were fixed via intravenous perfusion as described^5,27^. Harvested tissues underwent 4% PFA fixation and were subsequently transferred to pure ethanol. Lastly, ECi was used for clearing until completion (Sup. Fig. 1A).

Imaging was performed using the LaVision Ultramicroscope II, featuring a conventional configuration with excitation wavelengths ranging from 405 to 785 nm. The detailed configuration concerning excitation wavelength, emission filters and laser powers are provided in Supplementary Tables 1 and 2. To reduce acquisition artifacts in the individual images obtained under excitation from the left and the right side, image stacks were procured for each side of excitation individually and combined by digital image addition. Additionally, down sampling from an isotropic 5 µm to 20 µm resolution through trilinear averaging further reduced imaging artifacts (Sup. Fig. 1B).

In conclusion, we first established the standardized workflow in tissue samples of perfused mice to the reconstruction of the individual images, applicable for various organs and tissue structures.

### Ethyl cinnamate enables 3D imaging of albino mouse eye and surrounding soft tissues

**Figure 1:**
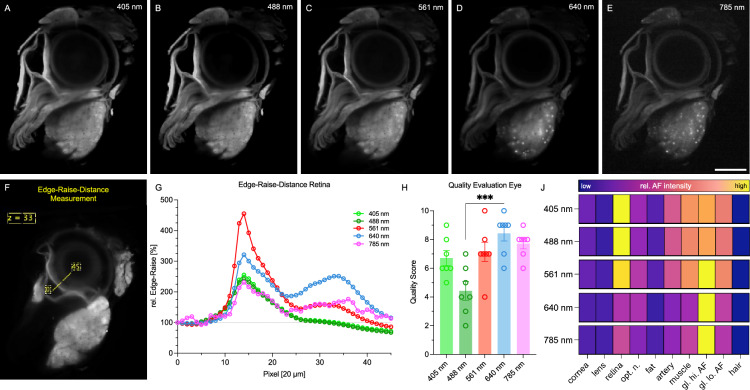
Autofluorescence characterization of different components of the murine eye. (**A**-**E**) Autofluorescence with varying excitation wavelength for 405 nm (**A**), 488 nm (**B**), 561 nm (**C**), 640 nm (**D**), 785 nm (**E**). Resolution adjusted to 20 µm. Scale bar 1 mm. (**F**) Scheme for the Edge-Raise-Distance quantification, slice number 33, from pixel 0–45. (**G**) Graphical results of the Edge-Raise-Distance for the complete AF spectra. (**H**) Subjective image quality evaluation. One data point per evaluator and channel. One-Way-ANOVA, *** *p* < 0.001. (**J**) Quantification of the relative AF characteristics of various anatomical regions (abbreviations: optic nerve (opt. n.), high autofluorescent portion of the lacrimal gland (gl. hi. AF), low autofluorescent portion of the lacrimal gland (gl. lo. AF)). Color bars indicating signal intensity to the right of the respective channel. Results display the mean from 3 independent animals.

Non-albino mice, such as C57BL/6, present a challenge due to melanin deposition, necessitating a bleaching step for successful ECi-based clearing of the eye^28^. In our developmental approach, we therefore sourced our samples from albino mice of BALB/c background. Employing an isotropic resolution of 20 µm, we identified and scrutinized ten distinct structures within the eye. We scanned the eye in all combinations of lasers and detectors possible with our microscope (Sup. Fig. 2). Notably, excitation lasers spanning 405–640 nm exhibited superior detail compared to 785 nm (Fig. 1A–E). Quantitative analysis involved measuring the Edge-Raise-Distance (ERD), defined as the pixel intensity values along a line within an image. ERD was deployed to quantify the transition from the retinal periphery to the lens across different laser lines (Fig. 1F–G). An expert panel of seven radiologists, collectively possessing over 20 years of experience, evaluated autofluorescence channels based on image quality. The 640 nm channel emerged as optimal for discerning ocular structures, while the 785 nm channel also gained high ratings, owing to minimal interference from artifacts. Conversely, the 488 nm channel received lower ratings likely due to a compromised signal-to-noise ratio and accentuated artifacts (Fig. 1H). Importantly, we detected highly autofluorescent particles attached to the sample during image transfer into the IMPAX software, particularly under 488 nm and 561 nm excitations, leading to overall image quality degradation as illustrated in Supplementary Fig. 3. Through quantitative analysis in Fiji, distinct AF spectra were observed for the retina, optic nerve, and specific punctate structures within the lacrimal gland (Fig. 1J).

### Assessing autofluorescence spectrum in musculoskeletal tissues

**Figure 2:**
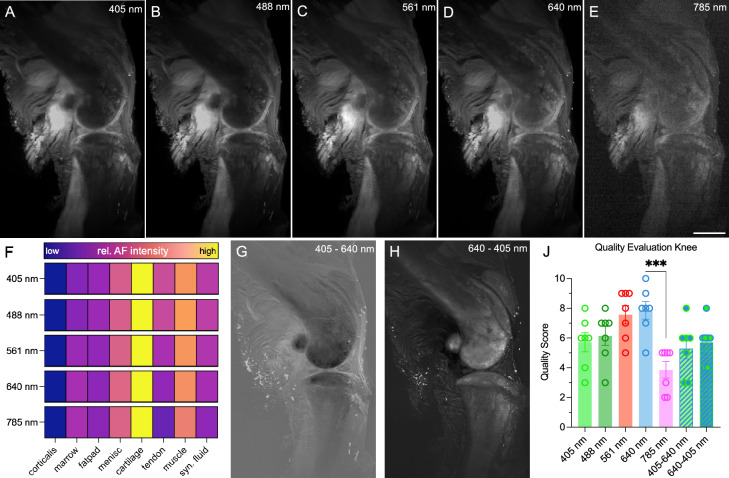
Autofluorescence characteristics of musculoskeletal tissue. (**A**–**E**) Autofluorescence with varying excitation wavelength for 405 nm (**A**), 488 nm (**B**), 561 nm (**C**), 640 nm (**D**), 785 nm (**E**). Resolution adjusted to 20 µm. Scale bar 1 mm. (**F**) Quantification of the relative AF characteristics of various anatomical regions (abbreviation: synovial fluid (syn. fluid)). Color bars indicating signal intensity to the right of the respective channel. Results display the mean from 3 independent animals. (**G**–**H**) Digital image subtraction of the 405 nm channel minus the 640 nm channel (**G**) and vice versa (**H**). (**J**) Subjective image quality evaluation. One data point per evaluator and channel. One-Way-ANOVA, *** *p* < 0.001.

In the exploration of a diverse range of tissue types, we employed the murine knee as a representative musculoskeletal sample. A fully detailed spectra of murine knee is present in Supplementary Fig. 4. In shallow tissue depths (i.e., shorter distance between the excited light sheet and the tissue surface facing the objective), short excitation wavelengths (405 nm, 488 nm, and 561 nm) provided preferable detail richness compared to longer wavelengths (640 nm and 785 nm) (Fig. 2A–E). We systematically characterized eight distinct anatomical regions, revealing cartilage as highly autofluorescent across all channels, with pronounced prominence in shorter wavelengths (Fig. 2F). As more substantial structures, such as bone, exhibited diminished transparency and light deflection toward the camera, image quality at 1 mm deeper tissue depths inversely correlated with excitation wavelength. Exploiting the disparities in autofluorescence spectra among the eight structures, we successfully enhanced cartilage and meniscus visibility through subtraction of the 640 nm channel from the 405 nm channel (Fig. 2G). The resulting inverted digital subtraction produced an image stack highlighting cortical and bone marrow regions (Fig. 2H), encompassing transcortical microvasculature, as described by others with elaborated staining protocols^29^. Evaluators overall rated the 640 nm channel highest, whereas digital subtraction images scored lower (Fig. 2J). The relatively lower scores for the digital subtraction images may be attributed to the fact, that only some tissue features are enhanced substantially at the expense of most others, conclusively reducing the overall quality perception.

### Distinctive autofluorescence pattern in murine liver

**Figure 3:**
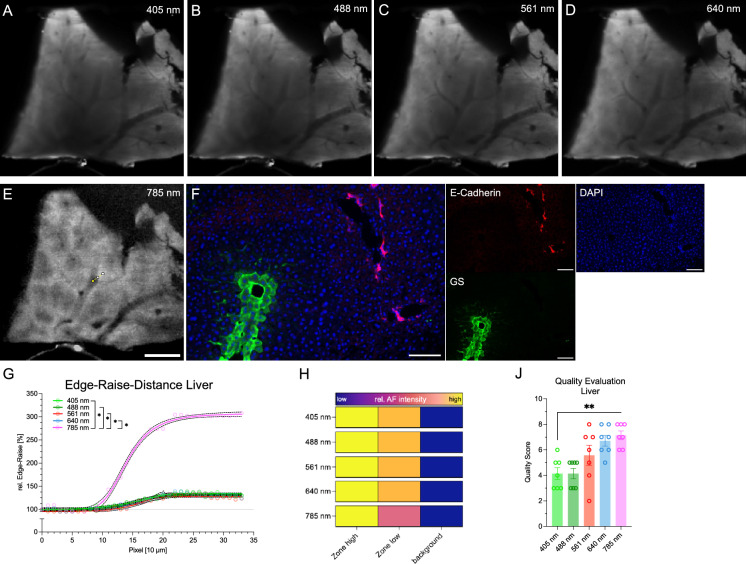
Autofluorescence of liver tissue. (**A**–**E**) Autofluorescence with varying excitation wavelength for 405 nm (**A**), 488 nm (**B**), 561 nm (**C**), 640 nm (**D**), 785 nm (**E**). Resolution adjusted to 20 µm. Scale bar 1 mm. Indicator for the Edge-Raise-Distance in (**E**), starting from yellow directing to white. (**F**) Representative Immunofluorescence of liver, scale bar 100 µm, 400x. (**G**) Representative Edge-Raise-Distance for all channels with dashed black lines for the 95% confidence interval. *p < 0.05. (**H**) Quantification of the relative AF characteristics of the low, high AF and background. Color bars indicating signal intensity to the right of the respective channel. Results display the mean from 4 independent animals. (**J**) Subjective image quality evaluation. One data point per evaluator and channel. One-Way-ANOVA, ** *p* < 0.01.

After exploration of the eye as well as musculoskeletal tissues we lastly utilized our pipeline towards a parenchymal abdominal sample. We scanned murine liver extensively with all possible combinations of lasers and detectors (Sup. Fig. 5). Our investigation of murine liver AF unveiled a unique spectral profile, evident across all priorly established channels and most pronounced at 785 nm excitation (Fig. 3A–E). The AF spectrum observed in the 785 nm channel was reminiscent of liver zonation, known from conventional immunofluorescence microscopy stained for glutamine synthetase (GS) and E-Cadherin (Fig. 3F). Strikingly divergent from the eye and knee structures studied earlier, statistical analysis of the ERD demonstrated a contrasting AF behavior, albeit deploying the same laser power and camera settings. Unlike the preceding structures, the pinnacle of relative AF did not align with excitation wavelengths ranging from 405 to 640 nm, instead manifesting at 785 nm (Fig. 3G–H). The evaluation by radiologists confirmed the highest information yield in the long wavelength excitation channels, with 785 nm ranking highest (Fig. 3J).

### Objective quantification of AF channel image sharpness

Next, we aimed to compare the findings regarding the channel image quality to an objective measure of image sharpness. We calculated the standard deviation of the power spectrum utilizing a Fourier analysis across all channels deployed for AF quantification (Sup. Fig. 6). Unexpectedly, the Fourier analysis determined the spectrum from 405 to 561 nm excitation superior to the 640 nm channel with the 785 nm channel exhibiting the lowest sharpness. These results indicate a stark difference between objective image sharpness and the quality/diagnostic value accredited by medical imaging professionals.

### Testing the compatibility of our protocol for antibody staining

**Figure 4:**
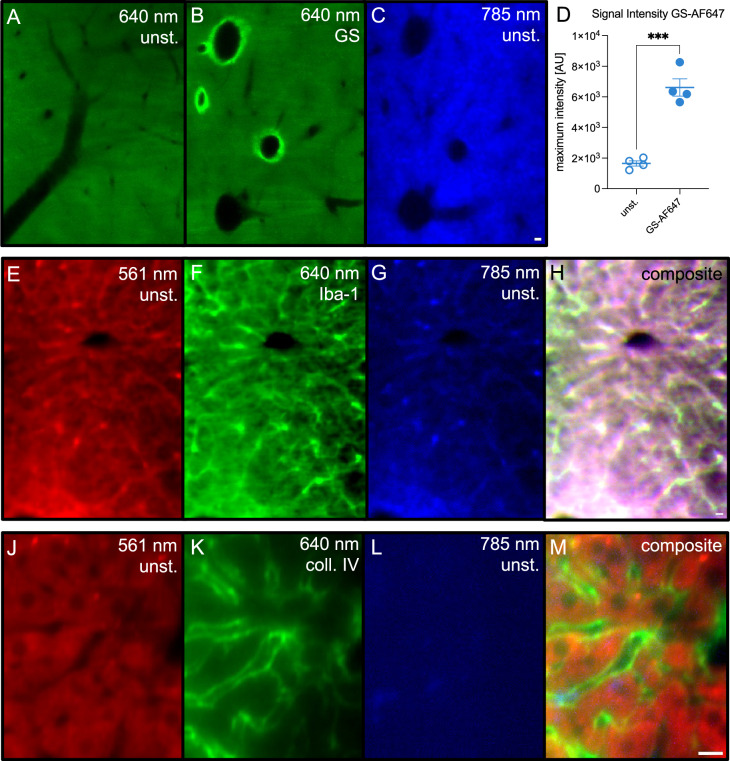
Evaluation of different antibody staining in combination with AF for liver tissue. (**A**–**C**) Test of anti-glutamine-synthetase-antibody in AF647 (GS), unstained control with excitation at 640 nm (**A**), staining with GS at 640 nm excitation (**B**) and the same sample as (**B**) at an excitation of 785 nm (**C**). (**D**) Increase in signal intensity in the 640 nm channel after staining with GS. (**E**–**G**) Test for anti-Iba-1-antibody in AF647 (Iba-1) in 561 nm (**E**), 640 nm (**F**), 785 nm (**G**) excitation wavelength. (**H**) Composite of (**E**–**H**). (**J**–**L**) Test for anti-collagen-IV-antibody in AF647 (coll. IV) in 561 nm (**J**), 640 nm (**K**), 785 nm (**L**) excitation wavelength. (**M**) Composite of (**J**–**L**). Scale bars in the bottom right of each row indicate 10 µm. Unpaired t-test for D with ***p < 0.01.

As liver tissue poses as a potential target for later application in percutaneous biopsies, we proceeded to test antibody based staining here (Fig. 4). First, we deployed an anti-glutamine-synthetase-AF647 (GS) staining and could sufficiently detect an increase in signal intensity around central veins in relation to the unstained control (Fig. 4A–D). However, some of the staining may overlap with the natural AF pattern in the 875 nm channel (Fig. 4C). To evaluate potential bleed through of fluorophore signals into AF channels, we next utilized anti-Iba1-AF647 (Iba-1) labeled antibody to mark macrophages in healthy liver tissue (Fig. 4E–H). The projection images show a severe bleed through into the 561 nm (Fig. 4E) and the 785 nm (Fig. 4G) AF channels. Lastly, we tested an anti-collagen-IV-AF647 (coll. IV) antibody in further liver samples (Fig. 4J–M). This particular antibody exhibited no quantifiable bleed through. Furthermore, we could demonstrate the high resolution capabilities of LSFM, as these image stacks were recorded with an isotropic resolution of 0.3 µm. Taken together, our findings demonstrate the principle feasibility for the combination of antibody-based staining with AF analysis, but likely the high epitope density of Iba-1 exposed a potential pitfall: There might exist a critical fluorophore concentration, in excess of which bleed through will corrupt the AF analysis of other channels.

### Machine learning-enhanced segmentation of unstained murine livers based on autofluorescence

**Figure 5:**
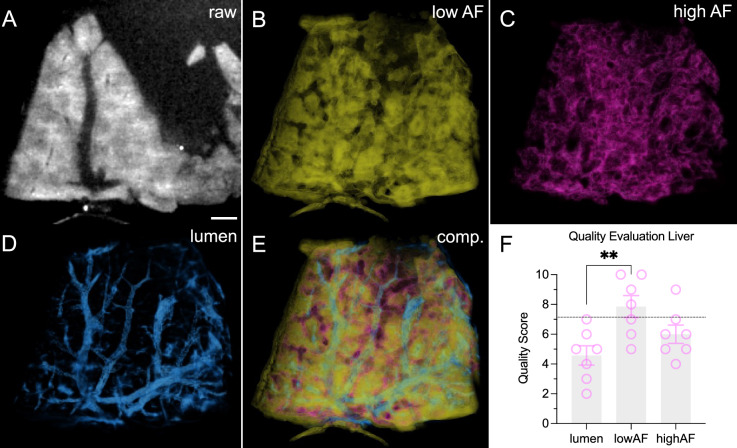
Machine Learning aids segmentation of unstained murine livers. (**A**) Raw image at 785 nm excitation. Scale bar 1 mm. (**B**–**E**) Machine learning generated probability maps for low autofluorescence areas (**B**), high autofluorescent areas (**C**), vasculature (**D**), composite (**E**). (**F**) Subjective image quality evaluation. One data point per evaluator and channel. Dotted horizontal line represents the score of the highest conventionally obtained liver image. One-Way-ANOVA, *p < 0.05.

Continuing the trajectory of advancing tissue assessment through AF, we postulated that machine learning could amplify the quality and diagnostic potential of raw images. To validate this concept, we selected the unstained liver—a tissue characterized by a relatively regular autofluorescence pattern—as the target for technical feasibility assessment (Fig. 5A). Employing an artificial intelligence framework called ‘Waikato Environment for Knowledge Analysis’ (WEKA), we trained the system using ten individual images derived from two livers under 785 nm excitation. Subsequently, we applied this trained framework to an independent sample. Ground truth data were obtained through manual delineation, demarcating image background, vascular structures, and high and low autofluorescent regions within the liver parenchyma. While WEKA can generate binary masks for each component class (data not shown), it can also generate probability maps for individual classes. The superior detail provided by probability maps led to their selection for subsequent analysis. WEKA-generated images effectively identified major components: the organ’s gross structure, represented by the inversion image of the background (Sup. Fig. 7), the low (Fig. 5B) and high AF regions (Fig. 5C) as well as the vasculature (Fig. 5D). A composite image for all WEKA segmented volumes is shown in Fig. 5E, adistinct composite for the low and high AF regions is provided as Supplementary Fig. 8 (detailed side-by-side presentation of each segmentation target as Sup. Video 1).

A panel of radiologists assessed both raw and WEKA-generated images regarding the overall image quality (Fig. 5F), with WEKA-generated images receiving the highest ratings. Notably, the low autofluorescence probability map was rated highest among all machine learning generated images, raising slightly above the highest score from a conventionally obtained channel (785 nm, with average score of 7.142). We also compared the WEKA generated liver zones with manually delineated image stacks (Sup. Fig. 9). The highly autofluorescent zone scored higher (DICE Score 0.82) than the low autofluorescent zone (DICE Score 0.42), which is likely due to a transitional zone not adequately distinguishable with our microscope.

### Simulating clinical percutaneous biopsies in mice

**Figure 6:**
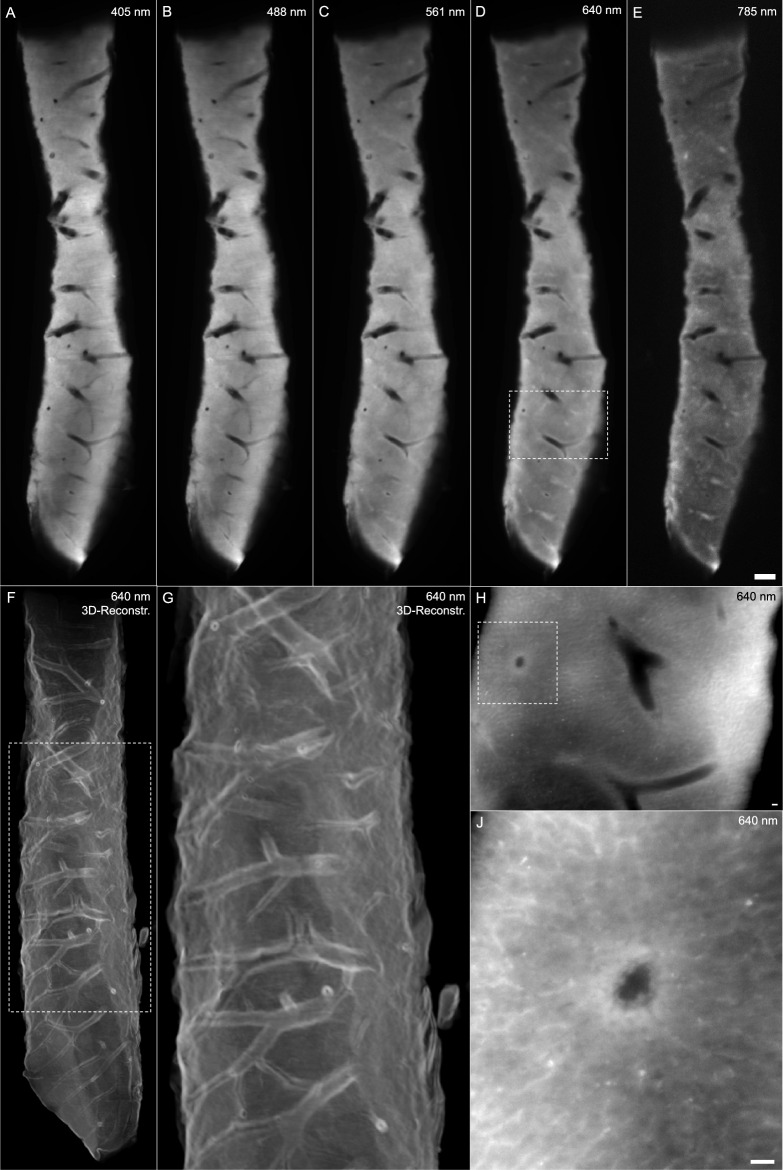
Simulated percutaneous liver biopsy in mice. (**A**–**E**) Projection images of the entire biopsy at 405 nm (**A**), 488 nm (**B**), 561 nm (**C**), 640 nm (**D**) and 785 nm (**E**). Scale bar in (**E**) represents 200 µm. (**F**) 3D surface rendition to visualize the vasculature and shape of the biopsy. Dashed rectangle shows the magnified image (**G**). (**H**) Images with a higher magnification objective in the 640 nm channel, dashed rectangle in (**D**) shows the field of view. The dashed rectangle in (**H**) indicates the field of view for the maximum resolution in (**J**). Scale bars in H and J represent 20 µm.

Conclusively, we tested our protocol in simulated percutaneous biopsies of parenchymal organs, i.e. liver. In this experiment, we sacrificed mice and opened the abdominal cavity to position the actual tissue extractor used in clinical practice, with no prior perfusion (Sup. Fig. 10). When triggered, the extractor’s integrated slit was released and cut out tissue cylinders with a maximum diameter of 1 mm (Sup. Video 2), much smaller than the tissue samples deployed in establishing our protocol. The overall sample quality was sufficient to delineate various anatomical details, as described above (Fig. 6A–E, Sup. Video 3). A surface rendition provided insight into the vasculature contained in the biopsy (Fig. 6F + G). Switching to a higher magnification objective, we could record the biopsy with an isotropic the resolution of up to 0.3 µm (Fig. 6H + J). Of note, this non-perfused sample exhibited remnants of blood, which exercises a relatively high AF in the far red spectrum (Sup. Fig. 11).

## Discussion

Light Sheet Fluorescence Microscopy (LSFM) has undergone remarkable advancements over the past decade, transforming our ability to visualize 3D biological structures in unprecedented detail^30–32^. These innovations have primarily focused on refining image quality, resolution, and the range of visualized markers by specific staining, permitting to image tissues of ever increasing dimensions^33^. This gave profound insights into tissue histology and pathology. While LSFM has predominantly been employed to address fundamental scientific questions, a growing interest in its clinical application is emerging. Notable progress has been made ranging from application of timely staining and imaging in human prostate biopsies to accentuating LSFM’s potential clinical utility^9^. In the future a possible integration of automated tissue processing devices, as described by Huang et al.^34^, is likely to have an enduring impact on the clinical transition of this innovative technology.

Contrary to the trend to optimize optical clearing aiming to image large samples, e.g. entire human organs, our study aimed to develop a workflow for percutaneous biopsies of parenchymal organs, which represent the vast majority of tissue samples harvested for diagnostic purposes. A simple, streamlined, and cost-efficient three-step optical clearing protocol post-tissue harvest, is crucial for clinical translation and applicability. Hence, we followed a radical approach: relying on the tissues AF exclusively and omitting all staining. Our hypothesis rested on the premise that the AF spectra of distinct tissue types would unveil distinctive patterns within and in between different tissues, rendering visual differentiation attainable.

Avoiding the mostly toxic and hazardous clearing substances used in scientific projects, we relied on ECi (after fixation with paraformaldehyde and dehydration via ethanol), a food additive approved by the European Food and Safety Authority^35^. This decision allowed us to perform the clearing and imaging without the need for fume hoods or extensive protective equipment. However, the use of ECi causes a limitation, which has to be kept in mind: Samples with pigmentation, foremost melanin, cannot be adequately imaged due to the ineffectiveness of ECi in optically clearing these structures. Future studies are warranted to establish sufficient bleaching mechanism compatible with our protocol.

Our LSFM was equipped with excitation lasers ranging from 405 to 785 nm, the associated conventional filter setups were chosen to enable the reproducibility of our work. We developed our protocol in three versatile tissue types: the intricate structures of the ocular globe (of albino mice) and its associated structures, the complex connecting tissues of the knee, and the parenchymal architecture of the liver. After developing AF spectral analysis for our protocol in LSFM, our study successfully delineated critical structures within the ocular globe and the murine knee. We observed a remarkable and reproducible variation in AF across different anatomical regions, offering potential avenues for enhancing image segmentation. Regarding the image quality, radiologists rated the 640 nm and 785 nm excitation wavelengths in tendency superior (with the exception of the 785 nm channel in the murine knee) to the shorter wavelengths. An un-biased analysis of image sharpness, however, found the shorter spectrum from 405 to 561 nm to generate more well-defined images. This might be due to the rather high background noise within the longer wavelength. Upcoming studies might aim to strengthen laser penetrance in the shorter wavelength by optimizing the optical clearing process. We further emphasize, that our measurements and comparisons via ERD require a standardized clearing protocol and microscopy setup to ensure robustness. In example, altering the laser power in one channel might alter the relations of the ERD measurements across different channels. Conclusively, future users are incentivized to thoroughly establish this workflow on their facilities.

Our protocol allows for additional staining with fluorophore-labeled antibodies. As the AF spectrum is rather dim by nature, as it is background, the laser power needed to image is higher than for stained samples. This might artificially contribute to bleed through of stained channels into an AF channel, as was the case for Iba-1 macrophage staining. Other antibodies may display a lower degree of bleed through as was the case for coll. IV and GS, which in turn enabled a combinational analysis of staining and AF. The underlying reason for bleed through might be a critical focal concentration of fluorophores, e.g. possibly due to a high Iba-1 epitope density. Our findings justify the testing of different labeling antibody concentrations in establishing a hybrid staining/AF analysis workflow in upcoming studies.

Particularly intriguing were the contrasting responses of the retina and cartilage to varying excitation wavelengths. This might present an opportunity for refining segmentation strategies and pinpointing region-specific markers just like equivalent approaches in radiology within the developing field of radiomics^36,37^. It is, however, important to note, that in this study we did not investigate any disease condition. We hypothesize that, upon disease, tissue AF is altered in a disease specific manner. Another aspect is the lack of human tissue in our study. However, to address the latter point, we simulated clinical percutaneous biopsies of the liver in non-perfused mice. Results indicate a feasibility of the proposed protocol. Further investigations are needed to test the AF spectrum in healthy and diseased patients.

The AF pattern observed in the retina was interesting for several further reasons, not directly related to the clinical applicability of the method though. Biopsies or extensive ex vivo analyses of the human eye are not part of clinical routine, as non-invasive techniques like fundus AF imaging are available^38^. Herein, the deposition of naturally or pathologically occurring intrinsic fluorophores are assessed. The dominant AF sources are fluorophores like lipofuscin and melanin^39^. In hindsight that albino mice were deployed here, this opens to consideration on what might be the composition of the high AF spots we identified.

Furthermore, our application of machine learning to AF spectral properties underscores their potential in driving more advanced analytical frameworks. To this end, our machine learning model primarily functions as a proof of principle rather than a directly applicable algorithm in human samples. Augmenting our results with machine learning procedures may hold promise for future studies regarding clinical applications. While prior works have harnessed AF properties for WEKA segmentation, our study extends its utility to 3D tissue analysis, potentially improving diagnostic procedures in the future^40^.

In conclusion, the synergy of a streamlined and cost-efficient optical clearing, AF spectral analysis, and machine learning confounds the translational potential of LSFM with exception to pigmented samples. This multifaceted approach not only expands the applicability in basic and biological sciences but also highlights the capability of LSFM in bridging the ‘resolution gap’ between histology and radiology.

## Methods

### Mice

BALB/c mice were bread in the animal facility of the iFET, University Hospital Bonn, Germany. Mice were housed with a 12 h day-night cycle and given food and water ad libitum. Mice were handled in accordance to the guidelines of the medical faculty at the University Bonn, all experimental protocols were approved by federal authorities (State Agency for Nature Conservation and Consumer Protection in North Rhine-Westphalia). The ARRIVE guidelines were followed.

### Light sheet fluorescence microscopy

Mice were sacrificed by exposure to gaseous CO_2_. Subsequently, mice were perfused first from the inferior vena cava and afterwards from the left cardiac atrium with 50 mM EDTA in phosphate buffered saline (PBS). Biopsies were acquired with a retractor (Quick-Core® Biopsy Needle 16G/9 cm, Ref: QC-16-9.0-20T) from a non-perfused mouse. Harvested organs and biopsies were incubated for 5 days at room temperature in 4% PFA 1% HEPES solution, on a rotator. After, the samples were changed to a 98% EtOH with 1% HEPES and incubated for 2 days at room temperature with rotation. Samples were lastly changed to ECi and incubated with rotation until fully cleared.

For stained samples, tissue was used after fixation and was incubated with a blocking buffer containing bovine serum albumin (BSA) and 1% goat normal serum (NGS) for 1 day at room temperature with shaking, incubated for 1 additional day with anti-GS (abcam, ab73593, 1:500), anti-Collagen IV (abcam, ab19808, 1:500), anti-Iba1 (abcam, ab178846, 1:500) in blocking buffer, and subsequently incubated with Alexa Fluor 647 conjugated goat anti-rabbit antibody (ThermoFisher, A-21244, 1:500) for 1 day. Tissue was washed in between steps with PBS containing 0.1% Triton-X-100. After staining, samples were incubated with 98% EtOH and cleared with ECi as described above.

Images were recorded with an Ultramicroscope II from LaVision Biotech with a raw image resolution of 2048 × 2048 pix. In all images, the numerical aperture of the microscope itself was set to 0.136 with a sheet thickness of 3.5 µm. A 1.3x (Type LVMI-Fluor, NA = 0.10, lateral resolution 5.0 µm) and a 12x (Type PLAN, NA = 0.53 DISCO, lateral resolution 0.6 µm without and 0.3 µm with inter-positioned magnification lens) ECi immersion objective were used. Step size was set to match the x- and y- lateral resolution. Excitation lasers 405 nm, 488 nm, 560 nm, 640 nm, and 785 nm were matched with the 525/50, 535/30, 620/20, 680/30, 845/55 filters, respectively. The exposure time for 405 nm, 488 nm, 560 nm, 640 nm was set to 104 ms, for 785 nm to 1040 ms (see Sup. Tabl. 1 + 2 for detailed information). Samples were excited with bilateral light-sheets, each side acquired individually and computed by simple addition using Fiji. Image series were processed with Fiji (Version 2.9.0/1.53t), 3D Slicer (Version 5.0.3), ParaView (Version 5.-11.0) and IMPAX (Version EE R 20 XIX SU1 v20200429_0936).

### Immunofluorescence microscopy

C57BL6J mice were sacrificed by CO_2_ exposure, perfused with PBS) and the liver was recovered and fixed in 4% PFA for at least 8 h. Samples were incubated with 30% sucrose in H_2_O for 1 day and embedded in OCT (Tissue-Tek, Sakura). 30 µm sections were cut on a Leica Cryostat and incubated for 1 h with blocking buffer (PBS, 5% BSA, 0.5% Triton-X (Sigma Aldrich), 1% NGS). Primary unconjugated antibody anti-GS (1:1000) was incubated overnight and secondary antibody (1:500) and E-Cadherin (clone 4A2, 1:100) and DAPI were incubated for 2 h. After wash, slides were mounted with ProLong Diamond mount (Thermo Fisher). Imaging was performed utilizing a Leica SP8 Lightning with a 40 × objective (Type HC PL APO, NA = 1.1 corr, water immersion).

### Radiological assessment of image quality

Images were initially reconstructed using Fiji by simple addition of the left and the right illumination images taken for each z-plane. In case for the knee joints, images were normalized by histogram and digitally subtracted to generate the 405–640 nm and the 640–405 nm virtual channels. In all cases, individual channels were exported as .tiff files. The DICOM export plugin in the program 3D slicer was utilized to translate the tiff files into DICOM format which in turn could be loaded into IMPAX (Agfa-Gevaert N.V.). In IMPAX, seven radiologists with over 20 years of experience were provided with each individual channel and multiplanar 3D reconstructions. Each radiologist was tasked to rate the overall image quality defined by richness of detail and absence of acquisition artifacts in a grade from 1 (poor quality) to 10 (high quality). The instructions given were the following: “A quality of 10/10 would be a perfect picture with not a single visible quality-flaw. At 5/10 you should still be able to obtain relevant medical information from the presented picture. At 2/10 and lower you cannot draw a medical conclusion in your opinion, either because the image is not sharp enough and/or significant portions of the image exhibit artifacts obstructing assessment.”

### Image augmentation using machine learning

The WEKA utility was used for a trainable AI procedure in Fiji, as previously described^41^. In our approach, liver data sets from the 785 nm channel at an isotropic resolution of 20 µm were used as input. Four classes were defined: Background, Lumen (referring to the entirety of vessels within the sample), Low (Autofluorescence) and High (Autofluorescence). Specific settings were: Field of view: max sigma = 16.0, min sigma = 0.0. Membrane thickness: 1, patch size: 19. The AI was manually trained using each five annotated images from two samples. A total of 228 attributes were calculated. Machine learning based image segmentation was carried out for an independent third liver sample. The resulting probability maps were postprocessed subtracting the ‘Background’ probability values from the other three image classes producing the definitive output.

### Supplementary Information

Supplementary Video 1.

Supplementary Video 2.

Supplementary Video 3.

Supplementary Information.

## Data Availability

Data underlying this study will be made available by the corresponding author upon reasonable request.

## References

[1] Richardson, D. S. & Lichtman, J. W. Clarifying tissue clearing. *Cell* **162**, 246–257. 10.1016/j.cell.2015.06.067 (2015). 26186186 10.1016/j.cell.2015.06.067 PMC4537058

[2] Molbay, M., Kolabas, Z. I., Todorov, M. I., Ohn, T. L. & Erturk, A. A guidebook for DISCO tissue clearing. *Mol. Syst. Biol.* **17**, e9807. 10.15252/msb.20209807 (2021). 33769689 10.15252/msb.20209807 PMC7995442

[3] Tainaka, K., Kuno, A., Kubota, S. I., Murakami, T. & Ueda, H. R. Chemical principles in tissue clearing and staining protocols for whole-body cell profiling. *Annu. Rev. Cell Dev. Biol.* **32**, 713–741. 10.1146/annurev-cellbio-111315-125001 (2016). 27298088 10.1146/annurev-cellbio-111315-125001

[4] Silvestri, L., Costantini, I., Sacconi, L. & Pavone, F. S. Clearing of fixed tissue: A review from a microscopist’s perspective. *J. Biomed. Opt.* **21**, 081205. 10.1117/1.JBO.21.8.081205 (2016). 27020691 10.1117/1.JBO.21.8.081205

[5] Klingberg, A. *et al.* Fully automated evaluation of total glomerular number and capillary tuft size in nephritic kidneys using lightsheet microscopy. *J. Am. Soc. Nephrol.* **28**, 452–459. 10.1681/ASN.2016020232 (2017). 27487796 10.1681/ASN.2016020232 PMC5280021

[6] Merz, S. F. *et al.* Contemporaneous 3D characterization of acute and chronic myocardial I/R injury and response. *Nat. Commun.* **10**, 2312. 10.1038/s41467-019-10338-2 (2019). 31127113 10.1038/s41467-019-10338-2 PMC6534576

[7] Kirschnick, N. *et al.* Rapid methods for the evaluation of fluorescent reporters in tissue clearing and the segmentation of large vascular structures. *iScience* **24**, 102650. 10.1016/j.isci.2021.102650 (2021). 34151237 10.1016/j.isci.2021.102650 PMC8192726

[8] Chatterjee, K., Pratiwi, F. W., Wu, F. C. M., Chen, P. & Chen, B. C. Recent progress in light sheet microscopy for biological applications. *Appl. Spectrosc.* **72**, 1137–1169. 10.1177/0003702818778851 (2018). 29926744 10.1177/0003702818778851

[9] Xie, W. *et al.* Prostate cancer risk stratification via nondestructive 3D pathology with deep learning-assisted gland analysis. *Cancer Res.* **82**, 334–345. 10.1158/0008-5472.CAN-21-2843 (2022). 34853071 10.1158/0008-5472.CAN-21-2843 PMC8803395

[10] Mallory, F. B. On certain improvements in histological technique: I. A differential stain for Amoebae Coli. II. phosphotungstic-acid-haematoxylin stain for certain tissue elements. III. A method of fixation for neuroglia fibres. *J. Exp. Med.* **2**(5), 529–533. 10.1084/jem.2.5.529 (1897). 19866848 10.1084/jem.2.5.529 PMC2132861

[11] Larsen, D. D., Gaudreault, N. & Gibbs, H. C. Reporting reproducible imaging protocols. *STAR Protoc.* **4**, 102040. 10.1016/j.xpro.2022.102040 (2023). 36861824 10.1016/j.xpro.2022.102040 PMC9996438

[12] Hou, Y. *et al.* Optogenetic control of background fluorescence reduction for CRISPR-based genome imaging. *Anal. Chem.* **94**, 8724–8731. 10.1021/acs.analchem.2c01113 (2022). 35666940 10.1021/acs.analchem.2c01113

[13] Siegmund, R., Werner, F., Jakobs, S., Geisler, C. & Egner, A. isoSTED microscopy with water-immersion lenses and background reduction. *Biophys. J.* **120**, 3303–3314. 10.1016/j.bpj.2021.05.031 (2021). 34246627 10.1016/j.bpj.2021.05.031 PMC8392127

[14] Ghithan, J. H. *et al.* Photobleaching reduction in modulated super-resolution microscopy. *Microscopy (Oxford)* **70**, 278–288. 10.1093/jmicro/dfaa062 (2021). 10.1093/jmicro/dfaa062 PMC13396269 33064828

[15] Royer, C. A. Fluorescence spectroscopy. *Methods Mol. Biol.* **40**, 65–89. 10.1385/0-89603-301-5:65 (1995). 7633532 10.1385/0-89603-301-5:65

[16] Yamaguchi, Y., Matsubara, Y., Ochi, T., Wakamiya, T. & Yoshida, Z. How the pi conjugation length affects the fluorescence emission efficiency. *J. Am. Chem. Soc.* **130**, 13867–13869. 10.1021/ja8040493 (2008). 18816053 10.1021/ja8040493

[17] Yan, L. *et al.* Fluorescence emission mechanism for the pi-conjugated zwitterion 2,4-Bisimidazolylphenol base on ESIPT: A TDDFT theoretical reconsideration. *Spectrochim. Acta A Mol. Biomol. Spectrosc.* **312**, 124043. 10.1016/j.saa.2024.124043 (2024). 38368821 10.1016/j.saa.2024.124043

[18] Donaldson, L. Autofluorescence in plants. *Molecules* **25**, 2393. 10.3390/molecules25102393 (2020). 32455605 10.3390/molecules25102393 PMC7288016

[19] Pegg, T. J., Gladish, D. K. & Baker, R. L. Algae to angiosperms: Autofluorescence for rapid visualization of plant anatomy among diverse taxa. *Appl. Plant. Sci.* **9**, e11437. 10.1002/aps3.11437 (2021). 34268017 10.1002/aps3.11437 PMC8272585

[20] Haddock, S. H., Moline, M. A. & Case, J. F. Bioluminescence in the sea. *Ann. Rev. Mar. Sci.* **2**, 443–493. 10.1146/annurev-marine-120308-081028 (2010). 21141672 10.1146/annurev-marine-120308-081028

[21] Achetib, N., Falkena, K., Swayambhu, M., Aalders, M. C. G. & van Dam, A. Specific fluorescent signatures for body fluid identification using fluorescence spectroscopy. *Sci. Rep.* **13**, 3195. 10.1038/s41598-023-30241-7 (2023). 36823309 10.1038/s41598-023-30241-7 PMC9950469

[22] Billinton, N. & Knight, A. W. Seeing the wood through the trees: A review of techniques for distinguishing green fluorescent protein from endogenous autofluorescence. *Anal. Biochem.* **291**, 175–197. 10.1006/abio.2000.5006 (2001). 11401292 10.1006/abio.2000.5006

[23] Leischner, U., Schierloh, A., Zieglgansberger, W. & Dodt, H. U. Formalin-induced fluorescence reveals cell shape and morphology in biological tissue samples. *PLoS ONE* **5**, e10391. 10.1371/journal.pone.0010391 (2010). 20436930 10.1371/journal.pone.0010391 PMC2861007

[24] Thavarajah, R., Mudimbaimannar, V. K., Elizabeth, J., Rao, U. K. & Ranganathan, K. Chemical and physical basics of routine formaldehyde fixation. *J. Oral Maxillofac. Pathol.* **16**, 400–405. 10.4103/0973-029X.102496 (2012). 23248474 10.4103/0973-029X.102496 PMC3519217

[25] Pinkert-Leetsch, D. *et al.* The murine male reproductive organ at a glance: Three-dimensional insights and virtual histology using label-free light sheet microcopy. *Andrology* **10**, 1660–1672. 10.1111/andr.13292 (2022). 36082398 10.1111/andr.13292

[26] Abadie, S. *et al.* 3D imaging of cleared human skin biopsies using light-sheet microscopy: A new way to visualize in-depth skin structure. *Skin Res. Technol.* **24**, 294–303. 10.1111/srt.12429 (2018). 29377352 10.1111/srt.12429

[27] Bohner, A. M. C. *et al.* Renal denervation exacerbates LPS- and antibody-induced acute kidney injury, but protects from pyelonephritis in mice. *J. Am. Soc. Nephrol.* **32**, 2445–2453. 10.1681/ASN.2021010110 (2021). 34599036 10.1681/ASN.2021010110 PMC8722799

[28] Henning, Y., Osadnik, C. & Malkemper, E. P. EyeCi: Optical clearing and imaging of immunolabeled mouse eyes using light-sheet fluorescence microscopy. *Exp. Eye Res.* **180**, 137–145. 10.1016/j.exer.2018.12.001 (2019). 30578790 10.1016/j.exer.2018.12.001

[29] Gruneboom, A. *et al.* A network of trans-cortical capillaries as mainstay for blood circulation in long bones. *Nat. Metab.* **1**, 236–250. 10.1038/s42255-018-0016-5 (2019). 31620676 10.1038/s42255-018-0016-5 PMC6795552

[30] Erturk, A. *et al.* Three-dimensional imaging of the unsectioned adult spinal cord to assess axon regeneration and glial responses after injury. *Nat. Med.* **18**, 166–171. 10.1038/nm.2600 (2011). 22198277 10.1038/nm.2600

[31] Cai, R. *et al.* Panoptic imaging of transparent mice reveals whole-body neuronal projections and skull-meninges connections. *Nat. Neurosci.* **22**, 317–327. 10.1038/s41593-018-0301-3 (2019). 30598527 10.1038/s41593-018-0301-3 PMC6494982

[32] Dvinskikh, L. *et al.* Remote-refocusing light-sheet fluorescence microscopy enables 3D imaging of electromechanical coupling of hiPSC-derived and adult cardiomyocytes in co-culture. *Sci. Rep.* **13**, 3342. 10.1038/s41598-023-29419-w (2023). 36849727 10.1038/s41598-023-29419-w PMC9970973

[33] Mai, H. *et al.* Whole-body cellular mapping in mouse using standard IgG antibodies. *Nat. Biotechnol.*10.1038/s41587-023-01846-0 (2023). 37430076 10.1038/s41587-023-01846-0 PMC11021200

[34] Huang, J. *et al.* A cationic near infrared fluorescent agent and ethyl-cinnamate tissue clearing protocol for vascular staining and imaging. *Sci. Rep.* **9**, 521. 10.1038/s41598-018-36741-1 (2019). 30679514 10.1038/s41598-018-36741-1 PMC6345820

[35] Additives, E. P. O. *et al.* Safety and efficacy of aryl-substituted primary alcohol, aldehyde, acid, ester and acetal derivatives belonging to chemical group 22 when used as flavourings for all animal species. *EFSA J.* **15**, e04672. 10.2903/j.efsa.2017.4672 (2017). 32625398 10.2903/j.efsa.2017.4672 PMC7010084

[36] Park, T. *et al.* Automated segmentation of the fractured vertebrae on CT and its applicability in a radiomics model to predict fracture malignancy. *Sci. Rep.* **12**, 6735. 10.1038/s41598-022-10807-7 (2022). 35468985 10.1038/s41598-022-10807-7 PMC9038736

[37] Poirot, M. G. *et al.* Robustness of radiomics to variations in segmentation methods in multimodal brain MRI. *Sci. Rep.* **12**, 16712. 10.1038/s41598-022-20703-9 (2022). 36202934 10.1038/s41598-022-20703-9 PMC9537186

[38] Calvo-Maroto, A. M. & Cervino, A. Spotlight on fundus autofluorescence. *Clin. Optom. (Auckl.)* **10**, 25–32. 10.2147/OPTO.S134637 (2018). 30214339 10.2147/OPTO.S134637 PMC6095574

[39] Schmitz-Valckenberg, S. *et al.* Fundus autofluorescence imaging. *Prog. Retin. Eye Res.* **81**, 100893. 10.1016/j.preteyeres.2020.100893 (2021). 32758681 10.1016/j.preteyeres.2020.100893 PMC12906268

[40] Strohl, F. *et al.* Label-free superior contrast with c-band ultra-violet extinction microscopy. *Light Sci. Appl.* **12**, 56. 10.1038/s41377-023-01105-6 (2023). 36864022 10.1038/s41377-023-01105-6 PMC9981877

[41] Arganda-Carreras, I. *et al.* Trainable Weka segmentation: A machine learning tool for microscopy pixel classification. *Bioinformatics* **33**, 2424–2426. 10.1093/bioinformatics/btx180 (2017). 28369169 10.1093/bioinformatics/btx180

