# Supplementary material for: Autofluorescence-based tissue characterization enhances clinical prospects of light-sheet-microscopy
Source: Sci Rep. 2024 Aug 4;14:18033. doi: 10.1038/s41598-024-67366-2 (PMC11298517; doi:10.1038/s41598-024-67366-2)
Supplement: Supplementary file 4 — Supplementary Information. [file 41598_2024_67366_MOESM4_ESM.pdf]

- Supplementary Material -

Autofluorescence-Based Tissue Characterization Enhances Clinical Prospects of Light-Sheet-Microscopy

Authors: Alice M. Jacob[1], Anna F. Lindemann[1], Julia Wagenpfeil[2], Sergej Geiger[2], Yannik C. Layer[2], Babak Salam[2], Sarah Panahabadi[3], Darius Kurt[2], Maximilian W. M. Wintergerst[4], Frank A. Schildberg[5], Daniel Kuetting[2], Ulrike I. Attenberger[2,6], Zeinab Abdullah[1,+], Alexander M. C. Böhner[2,+,*]

1 Institute of Molecular Medicine and Experimental Immunology, University Hospital Bonn, Bonn, Germany.
2 Clinics for Diagnostic and Interventional Radiology, University Hospital Bonn, Bonn, Germany.
3 Clinic for Diagnostic and Interventional Neuroradiology, University Hospital Bonn, Bonn, Germany.
4 Department of Ophthalmology, University Hospital Bonn, Bonn, Germany.
5 Clinic for Orthopedics and Trauma Surgery, University Hospital Bonn, Bonn, Germany.
6 Department of Biomedical Imaging and Image-guided Therapy, Medical University of Vienna & General Hospital, Vienna, Austria.

+ These authors contributed equally
* Corresponding author: alexander.boehner@ukbonn.de

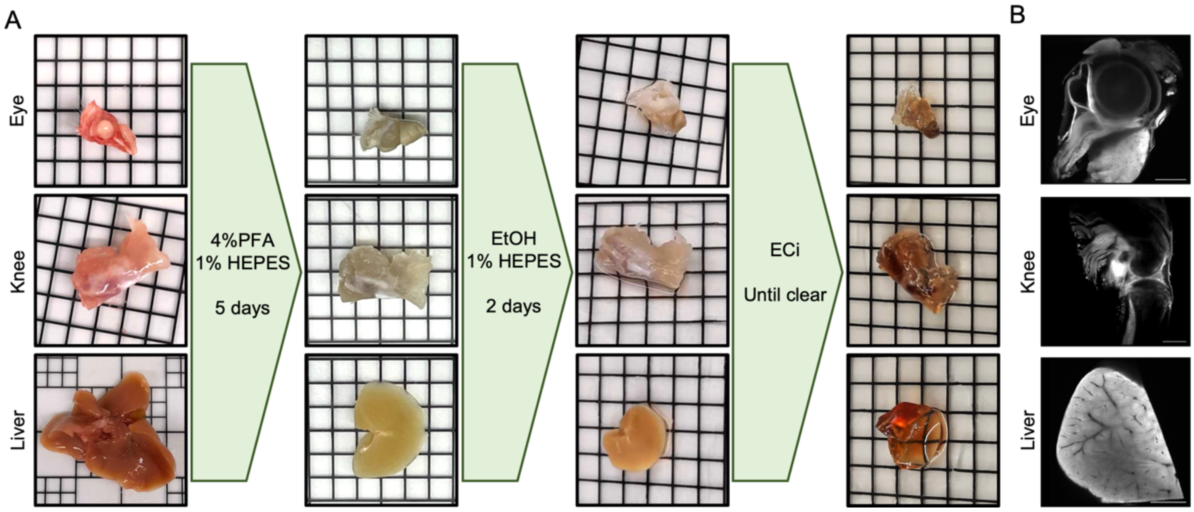

Sup. Fig. 1: Optical Clearing procedure and raw image aspect

A) Photographic images of an eye (top row), of a knee joint with connecting tissue (middle row) and of a liver lobe (bottom row). Samples directly after explantation in the left columns, and throughout the optical clearing procedure with the finalized result in the 4th column. One small square is ca. 2 mm. B) Overview images at 561 nm excitation reflecting for the respective samples. Scale bar in the bottom right is 1 mm.

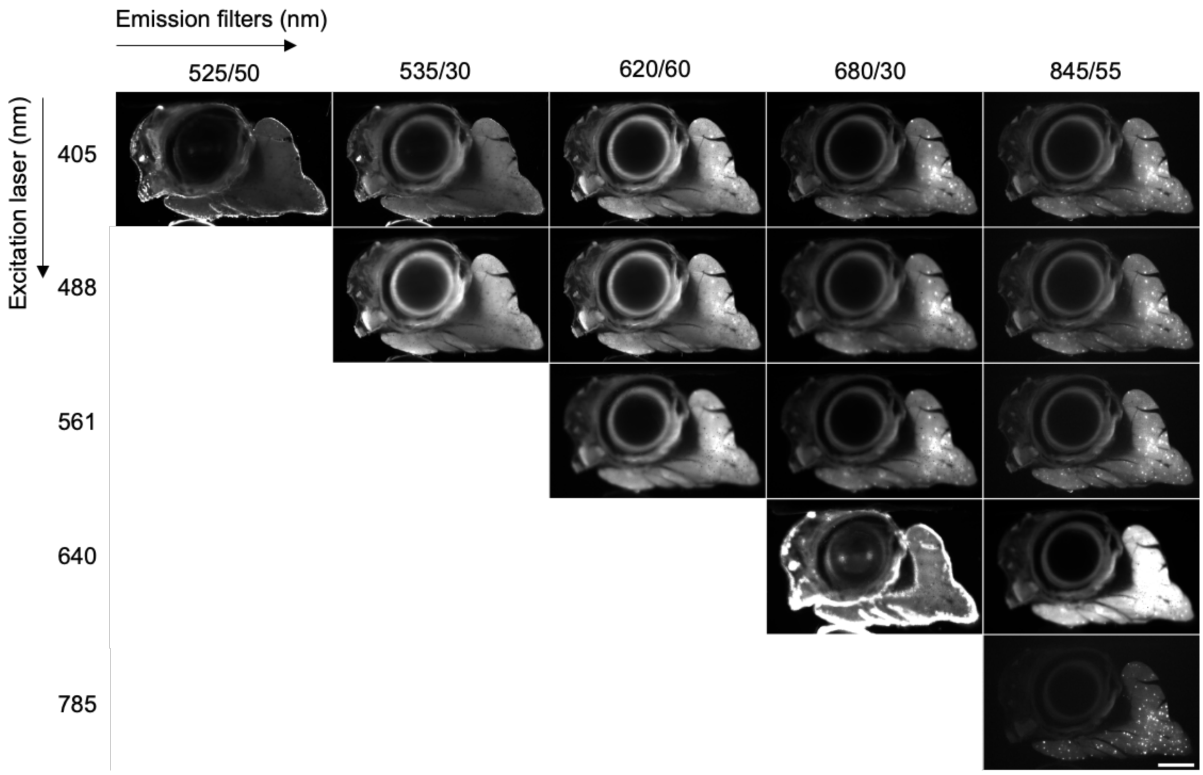

Sup. Fig. 2: Imaging of the murine eye with all laser / filter combinations

No artifact in plane

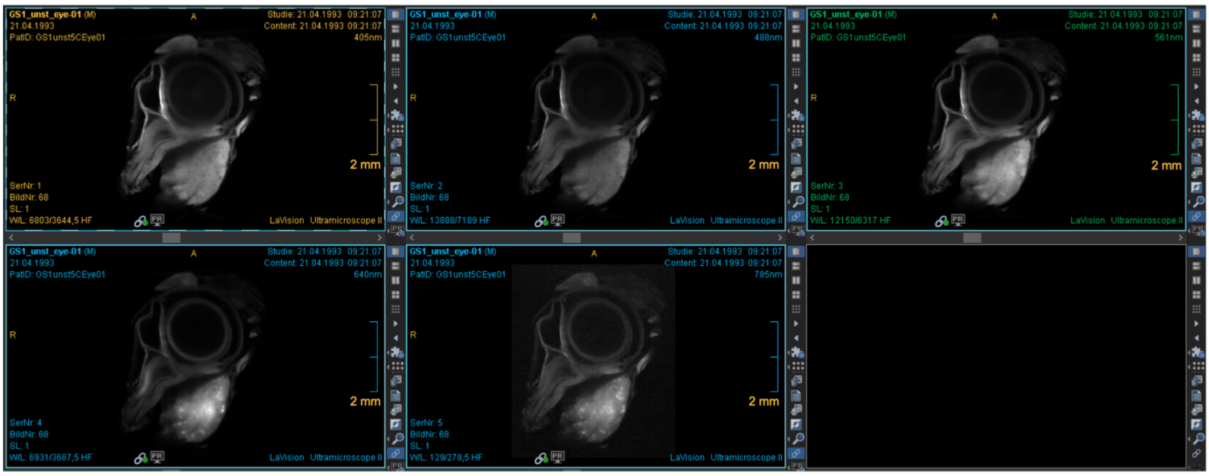

Artifact in plane (yellow arrow)

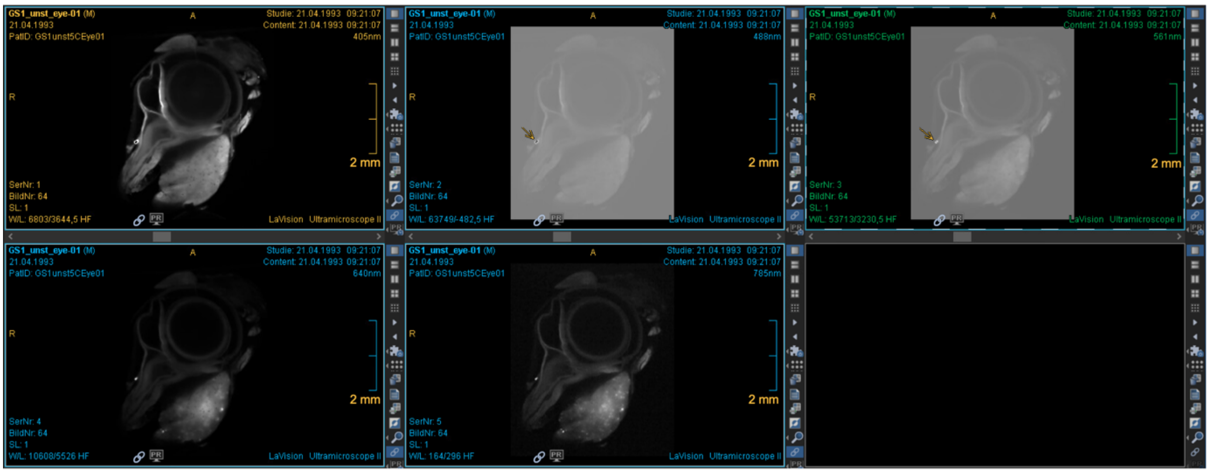

Sup. Fig. 3: Representation of the eye in IMPAX

Top: Tiles show different wavelengths from 405 nm to 785 nm. As title (No artifact in plane) indicates, no highly autofluorescent artifacts are present in this particular frame. Bottom: Artifacts in the 488 nm and the 561 nm channel (yellow arrows) indicating the location of highly autofluorescent particles corrupting the aspect of the entire frame. Note: The particle is also present in the other channels, but does not lead to overall file corruption. Scale bar in the right marks 2 mm.

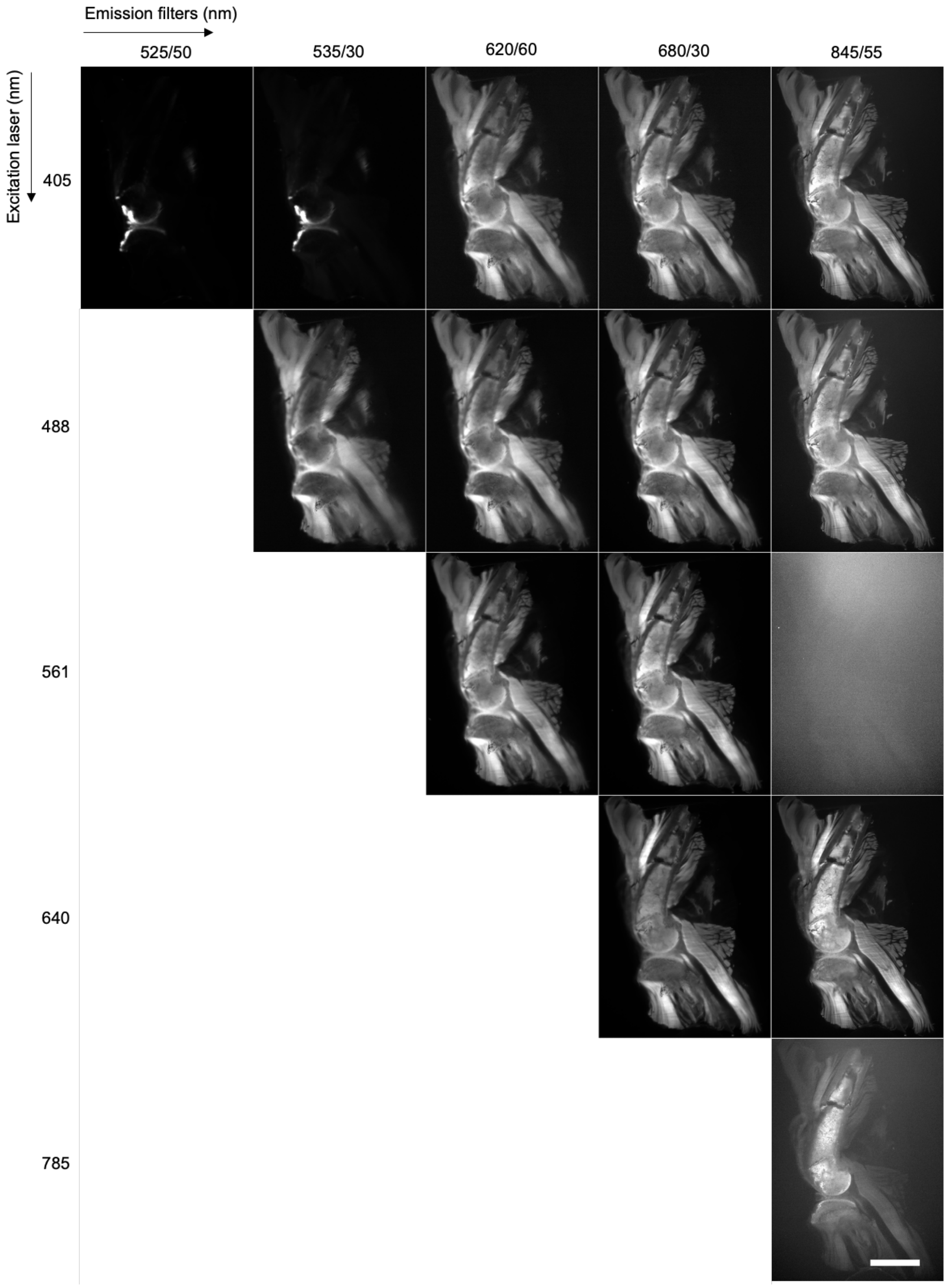

Sup. Fig. 4: Imaging of the murine knee with all laser / filter combinations

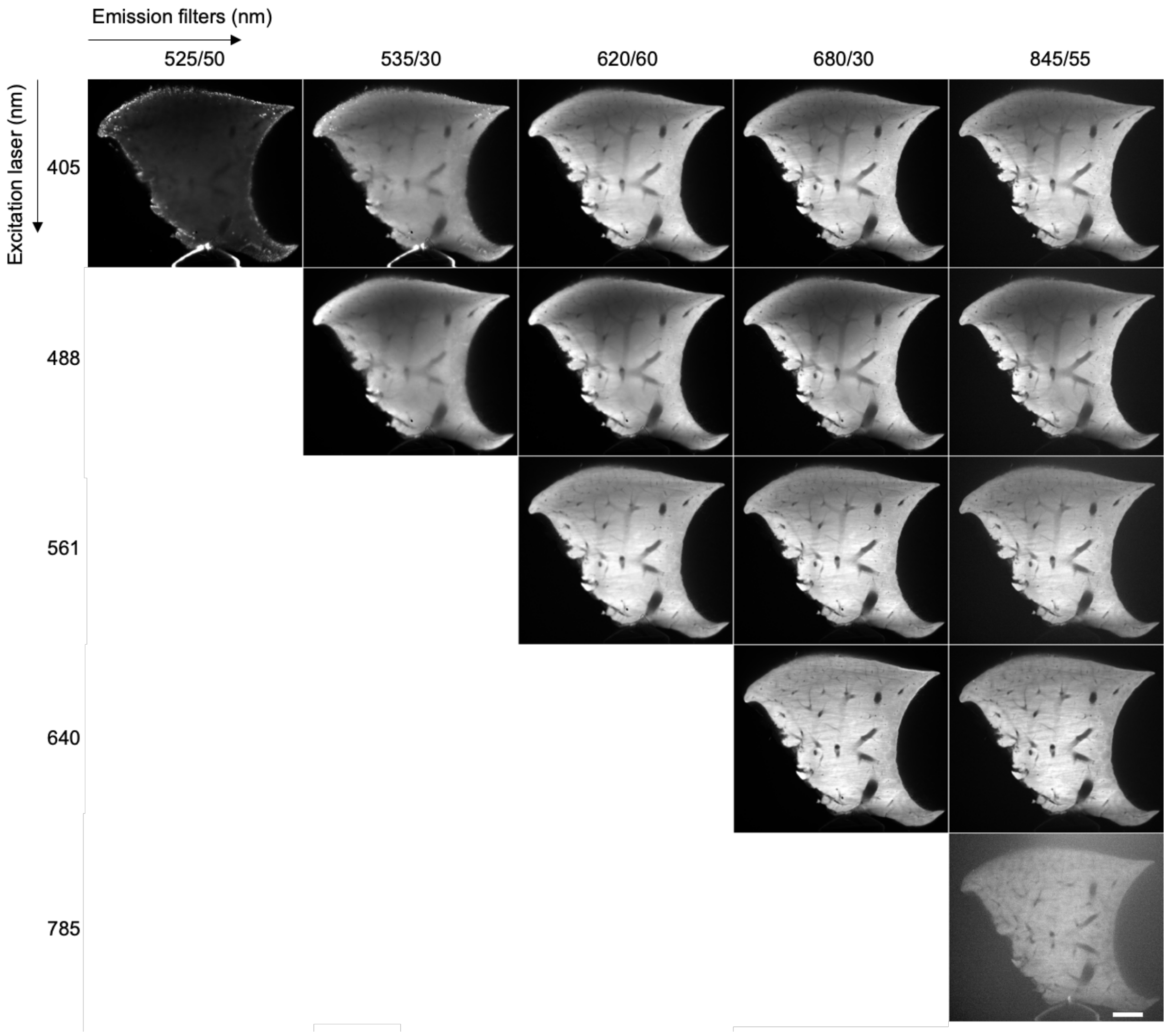

Sup. Fig. 5: Imaging of the murine liver with all laser / filter combinations

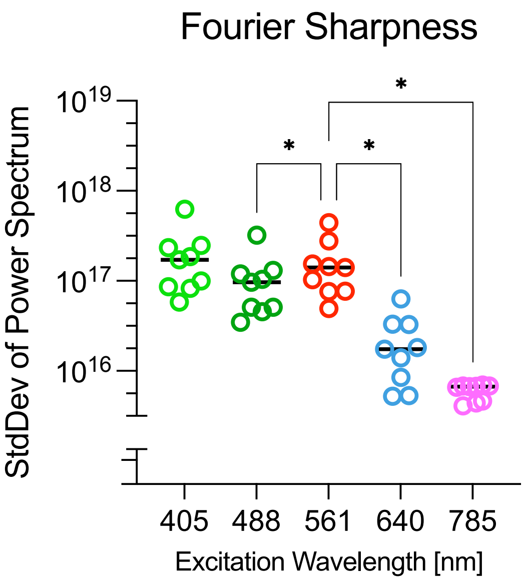

Sup. Fig. 6: Computational quantification of image sharpness
For each 3 eyes, knees and liver according to the specific excitation wavelength as standard deviation of the power spectrum generated by a Fourier image analysis. One-Way-ANOVA, * $p < 0.05$.

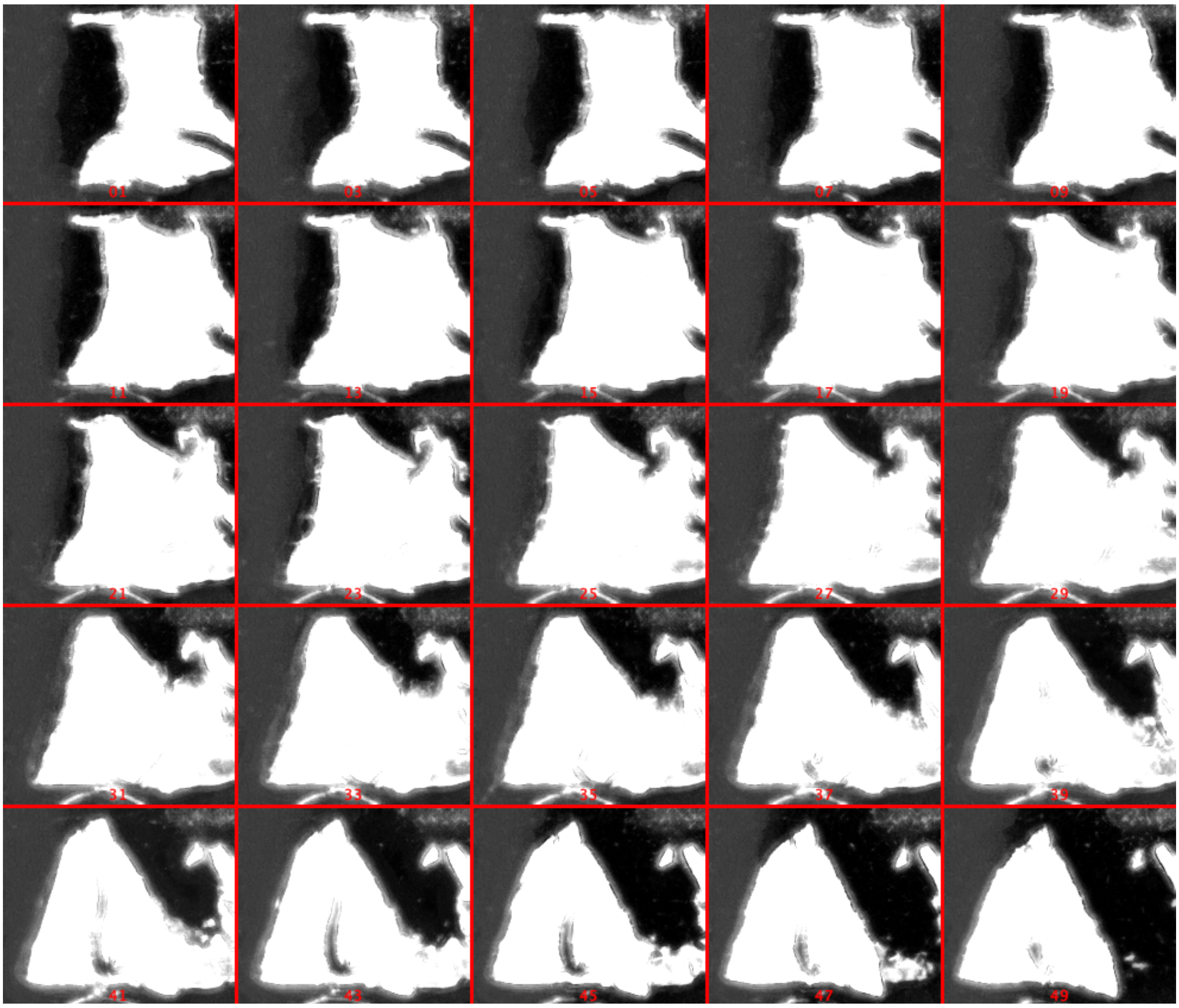

Sup. Fig. 7: WEKA segmentation result for organ shape (white) as an inversion of the area outside the sample (black and dark grey)

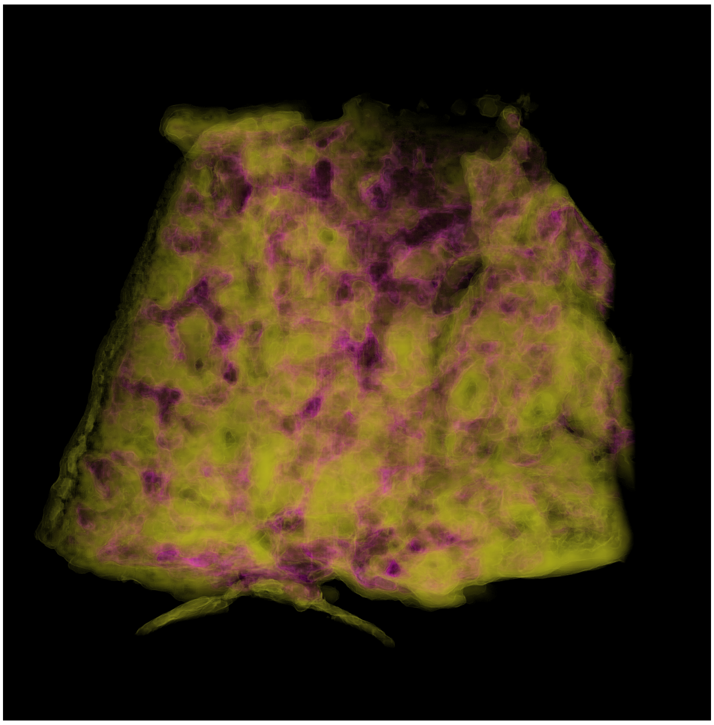

Sup. Fig. 8: WEKA segmentation results for the liver parenchyma with low AF areas in yellow and high AF areas in purple

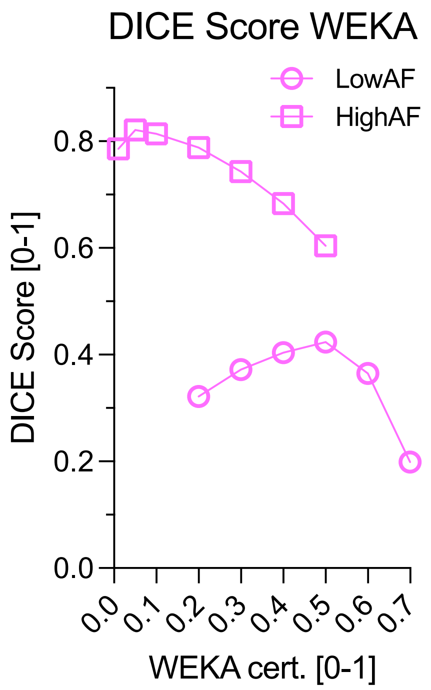

Sup. Fig. 9: DICE Similarity Coefficient for low and high autofluorescent liver zones in comparison to WEKA generated probability maps

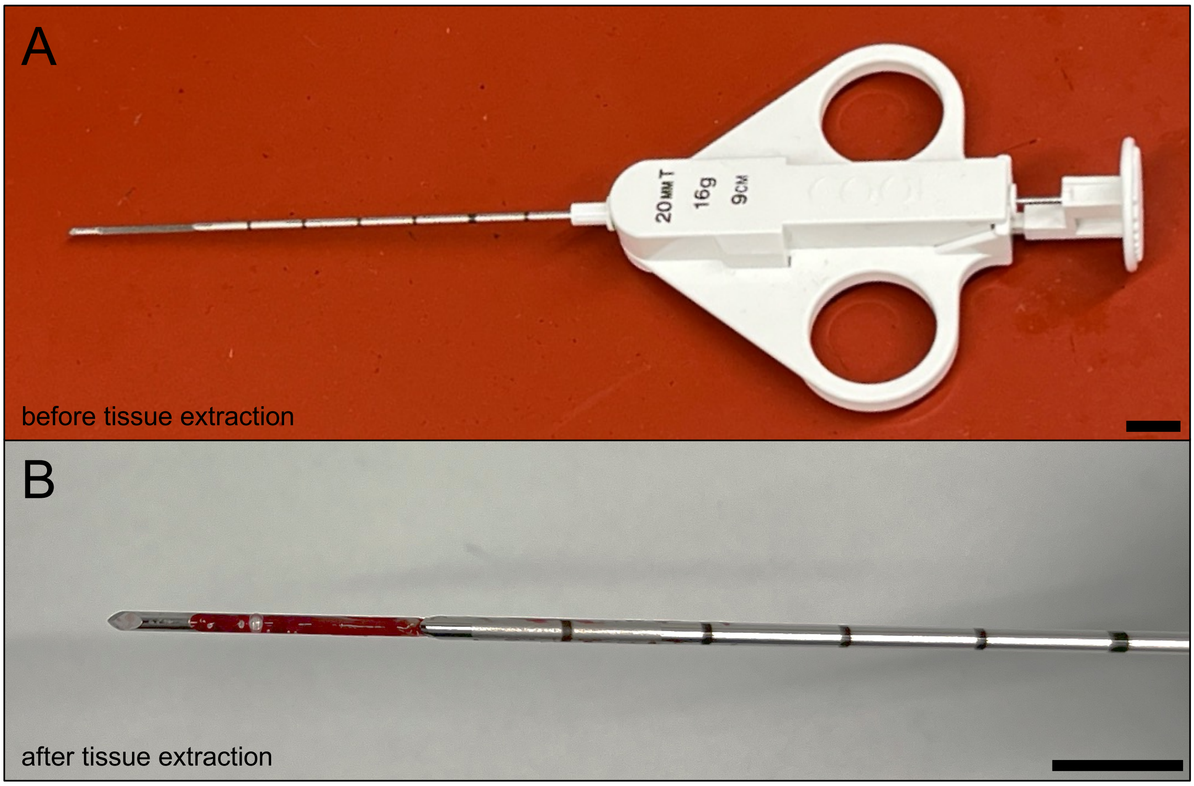

Sup. Fig. 10: Tissue sample collector before (A) and after tissue extraction (B). Scale bars represent 1 cm

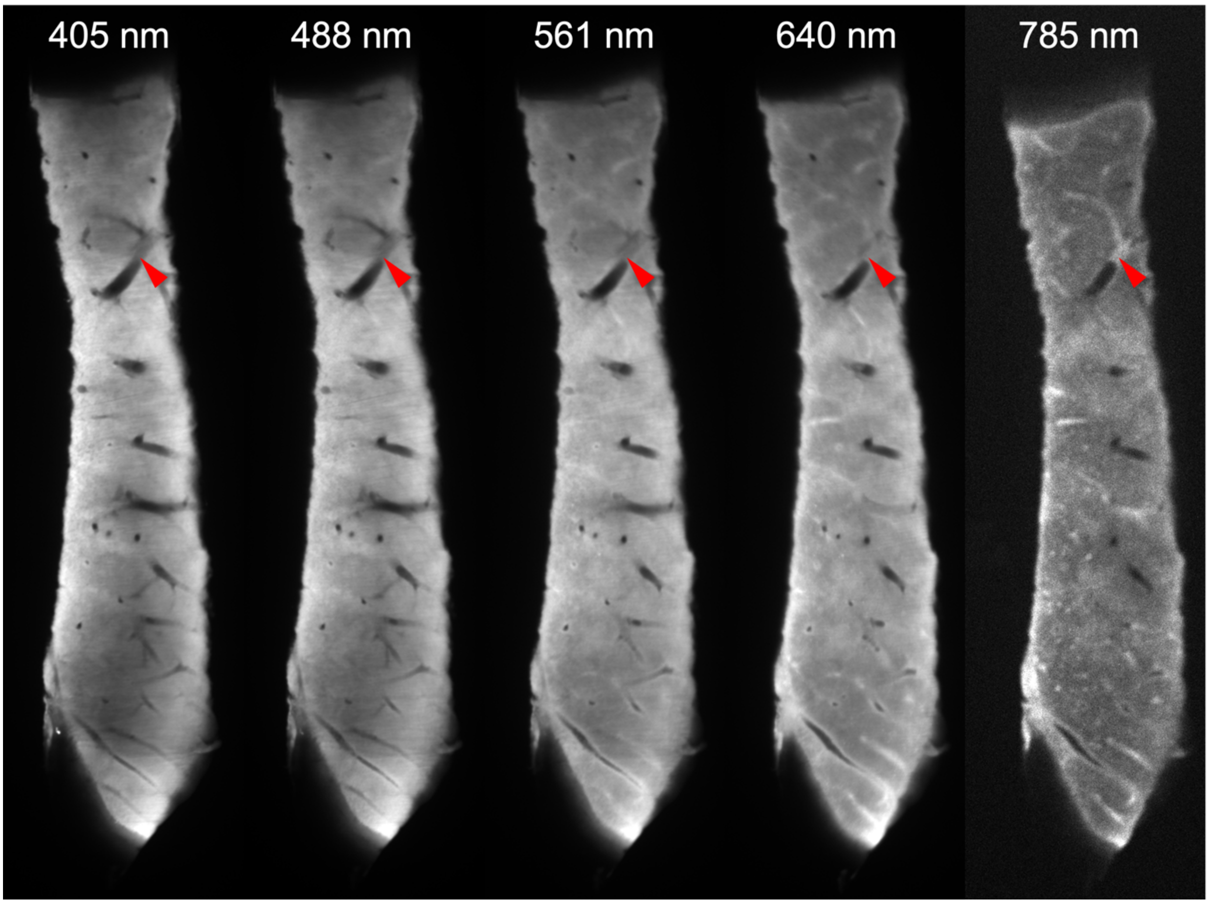

Sup. Fig. 11: Blood residual (marked with red arrowheads) in the simulated liver biopsy across all channels.

Excitation wavelength [nm]	Emission filter [nm]	LASER power [%]	Exposure time [ms]	Excitation wavelength [nm]	Emission filter [nm]	LASER power [%]	Exposure time [ms]
405	525 / 50	1	1500 (Knee: 50)	640	525 / 50	20	1500 (Knee: 50)
405	535 / 30	3	1500 (Knee: 50)	640	535 / 30	20	1500 (Knee: 50)
405	620 / 20	3	1500 (Knee: 50)	640	620 / 20	1	1500 (Knee: 50)
405	680 / 30	10	1500 (Knee: 50)	640	680 / 30	10	1500 (Knee: 50)
405	845 /55	35	1500 (Knee: 2500)	640	845 /55	30	1500 (Knee: 50)
488	525 / 50	2	1500 (Knee: 50)	785	525 / 50	10	1500 (Knee: 50)
488	535 / 30	3	1500 (Knee: 50)	785	535 / 30	20	1500 (Knee: 50)
488	620 / 20	3	1500 (Knee: 50)	785	620 / 20	1	1500 (Knee: 50)
488	680 / 30	3	1500 (Knee: 2500)	785	680 / 30	50	1500 (Knee: 50)
488	845 /55	15	1500 (Knee: 50)	785	845 /55	50	1500 (Knee: 50)
561	525 / 50	20	1500 (Knee: 50)				
561	535 / 30	25 (Liver: 20)	1500 (Knee: 50)				
561	620 / 20	5	1500 (Knee: 50)				
561	680 / 30	10	1500 (Knee: 50)				
561	845 /55	20	1500 (Knee: 50)				

Sup. Table 1: Ultramicroscope Setup for exploratory measurements

Excitation wavelength [nm]	Emission filter [nm]	LASER power [%]	Exposure time
405	525 / 50	15	104
488	535 / 30	25	104
561	620 / 20	15	104
640	680 / 30	35	104
785	845 /55	50	1040

Sup. Table 2: Ultramicroscope Setup for extensive AF characterization

Sup. Video 1: Video of the WEKA segmentation results (low AF areas in yellow, high AF areas in purple, vasculature in blue, composite image in the bottom right)

Sup. Video 2: Video of a simulated liver biopsy with the tissue collector used in clinical practice.

Sup. Video 3: Video of the image stacks for a simulated liver biopsy. This file corresponds to Figure 6A-E and displays the excitation wavelengths 405 nm, 488 nm, 561 nm, 640 nm and 785 nm from left to right.
